# Supplementary material for: Integrated Metabolomic and Transcriptomic Analysis Reveals Differential Mechanism of Flavonoid Biosynthesis in Two Cultivars of Angelica sinensis
Source: Molecules. 2022 Jan 4;27(1):306. doi: 10.3390/molecules27010306 (PMC8746331; doi:10.3390/molecules27010306)
Supplement: Supplementary file 1 [file molecules-27-00306-s001.zip › molecules-1506768-SI.pdf]

Supplemental Materials

Integrated Metabolomic and Transcriptomic Analysis Reveals Differential Mechanism of Flavonoid Biosynthesis in Two Cultivars of *Angelica sinensis*

Tiantian Zhu [1,2], Minghui Zhang [1], Hongyan Su [3], Meiling Li [3], Yuanyuan Wang [1,2], Ling Jin [1,2,*] and Mengfei Li [3,*]

[1] College of Pharmacy, Gansu University of Chinese Medicine, Lanzhou 730101, China; ztt0935@gszy.edu.cn (T.Z.); zmh0819@tom.com (M.Z.); wyy19880821@126.com (Y.W.)
[2] Northwest Collaborative Innovation Center for Traditional Chinese Medicine, Lanzhou 730000, China
[3] State Key Laboratory of Aridland Crop Science, Gansu Agricultural University, Lanzhou 730070, China; Shy922322@163.com (H.S.); mlli1996@163.com (M.L.)
* Correspondence: jinl@gszy.edu.cn (L.J.); lmf@gsau.edu.cn (M.L.)

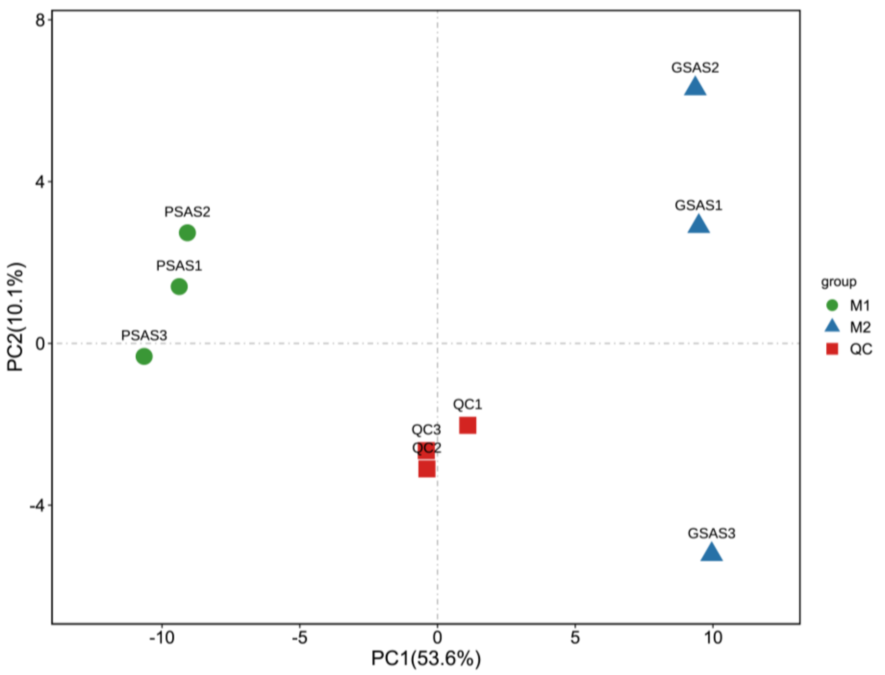

Figure S1: PCA of M1 (PSAS) and M2 (GSAS) as well as quality control (QC) samples.

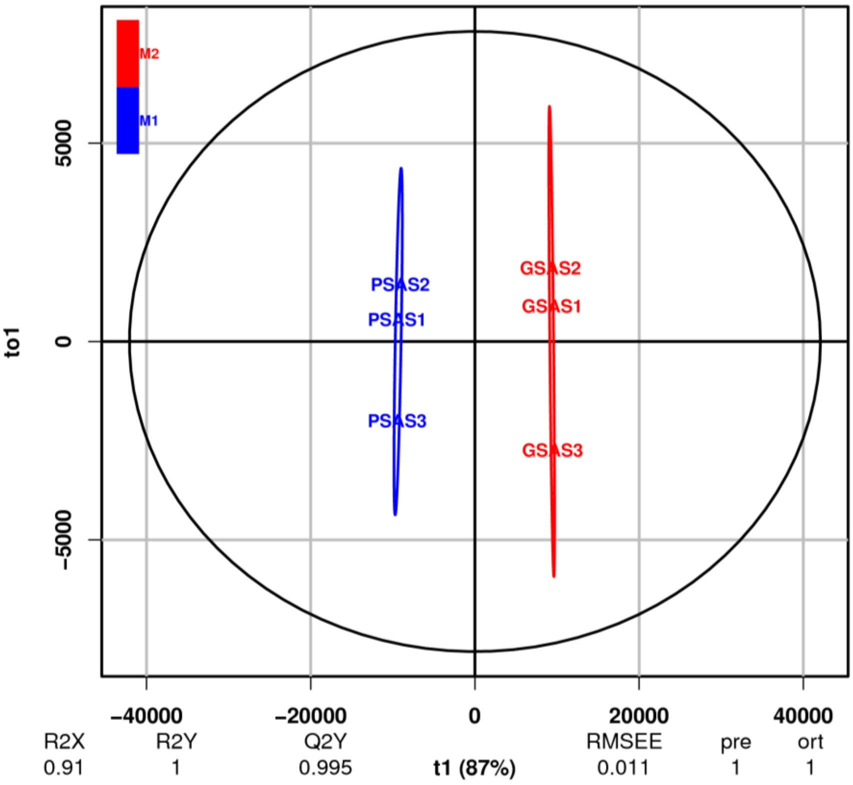

Figure S2: OPLS-DA of M1 and M2.

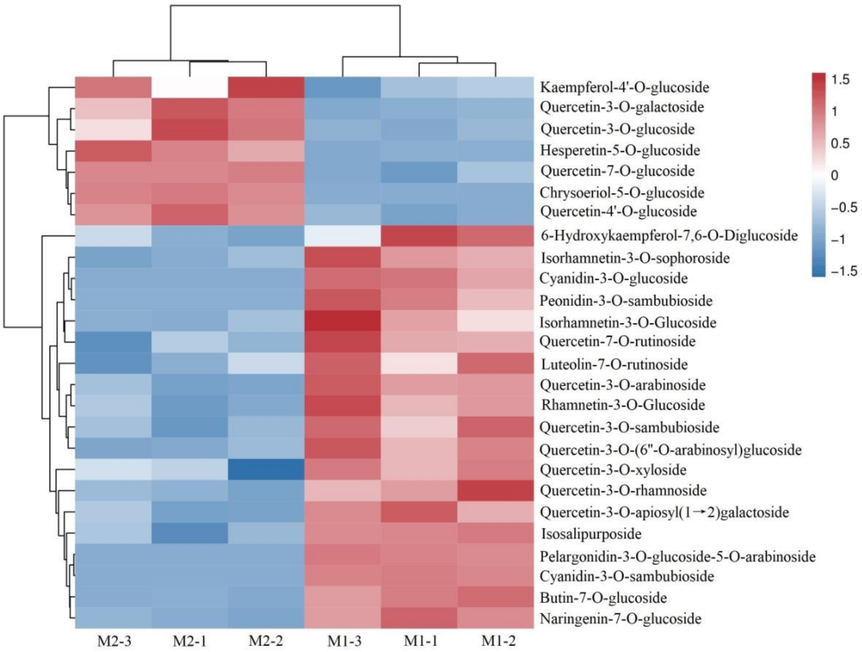

Figure S3: Cluster heat map of the 26 DAFs in M1 vs M2.

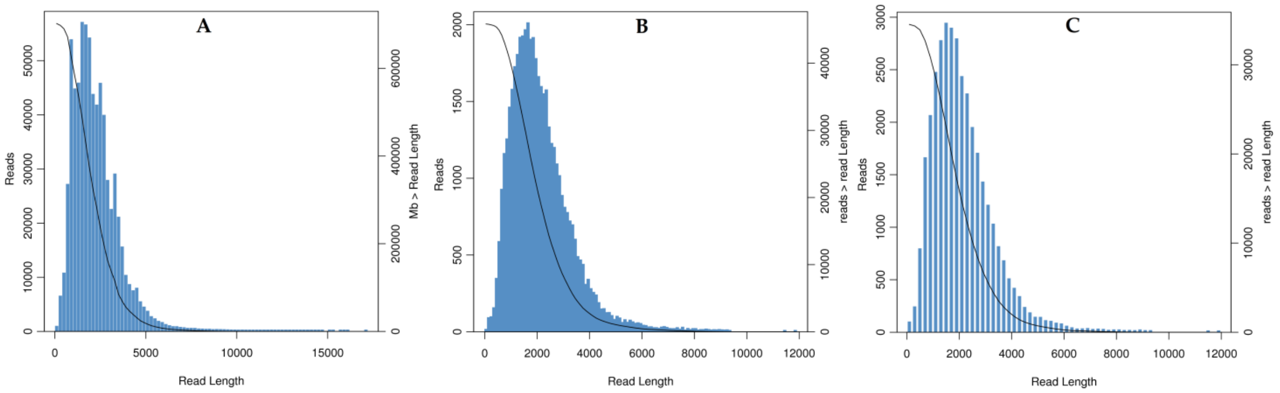

Figure S4: Length distribution of high-fidelity reads (A), high-quality isoforms (B) and full-length isoforms (C).

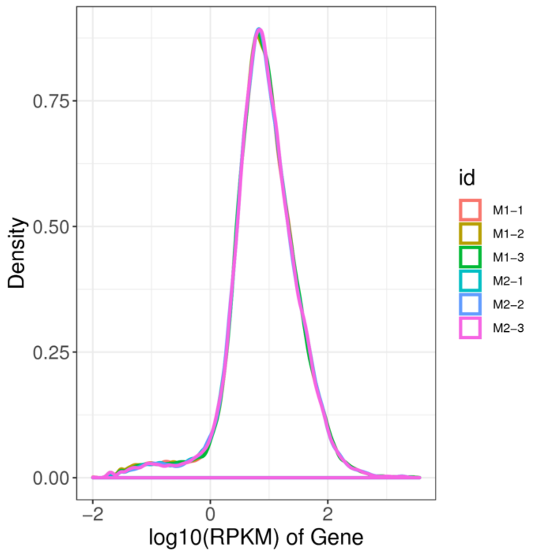

Figure S5: RPKM distribution of M1 and M2.

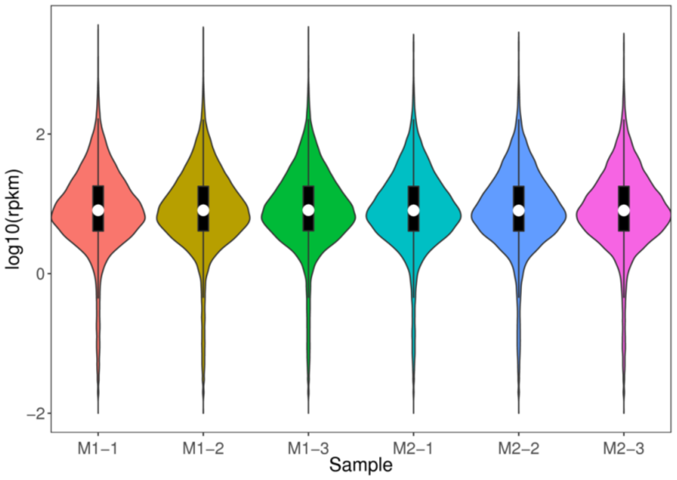

Figure S6: Violin plot of expression in M1 and M2.

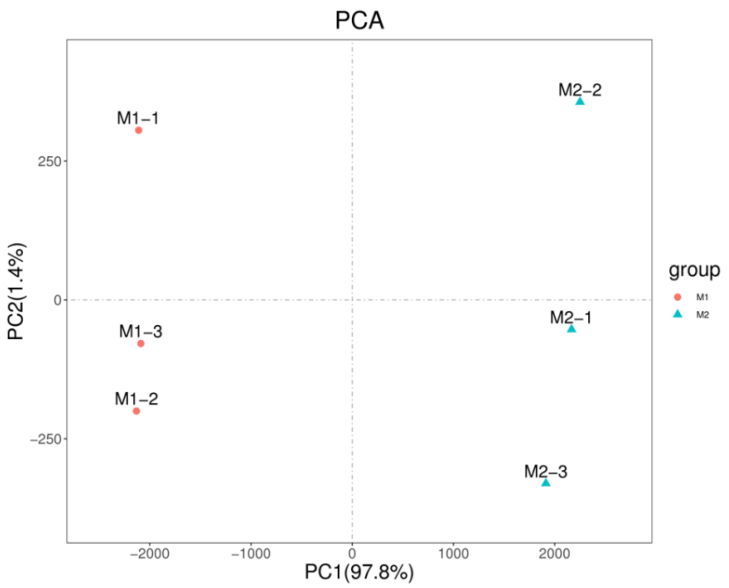

Figure S7: PCA analysis of M1 and M2.

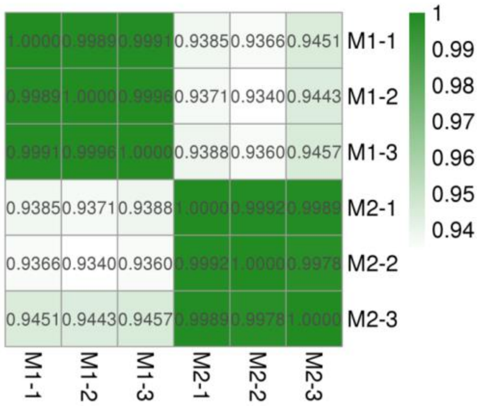

Figure S8: Pearson Heat-map correlation between M1 and M2.

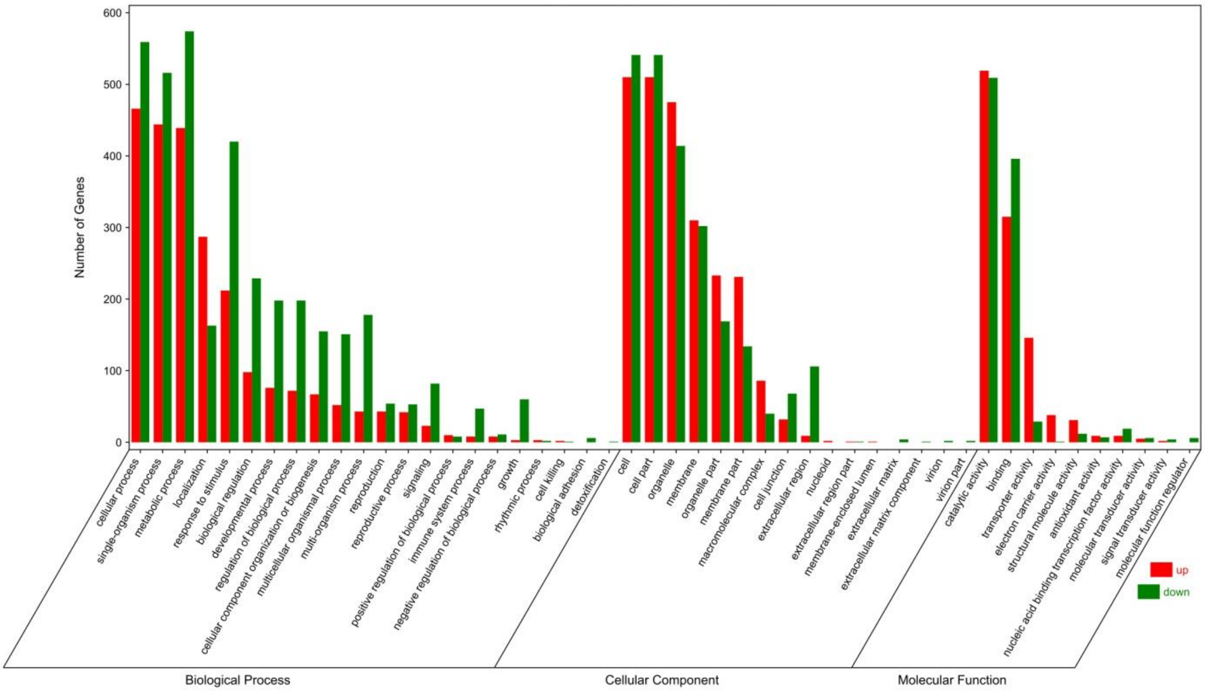

Figure S9: GO classification of the DEGs in M1 vs M2.

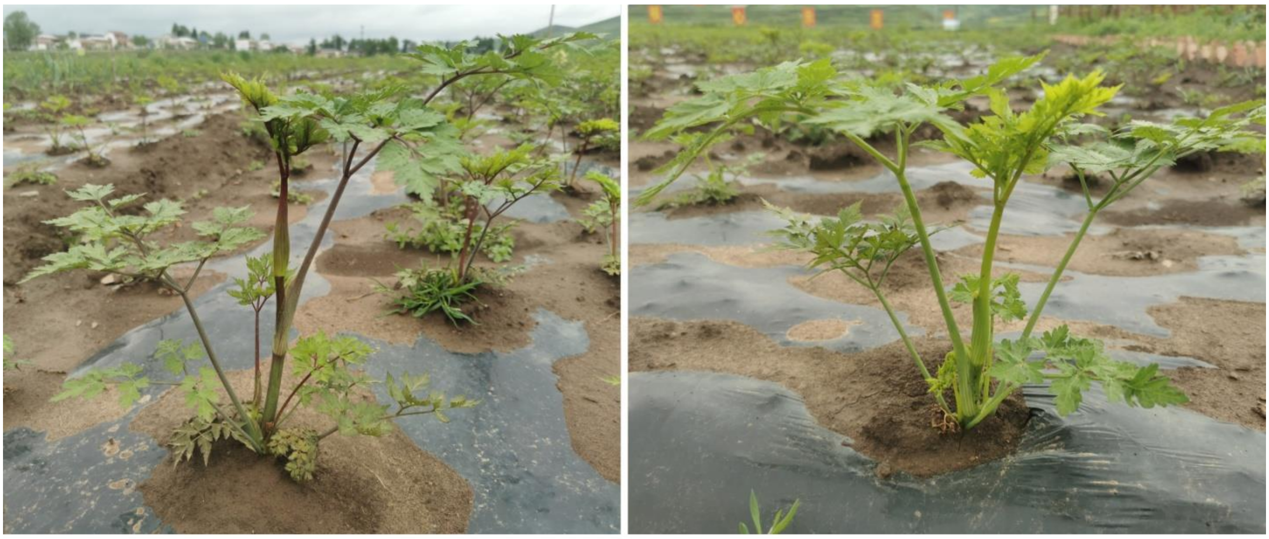

Mingui 1 (M1) with purple stem **Mingui 2 (M2) with green stem**

Figure S10: Aerial-parts characteristics of the two *Angelica sinensis* cultivars: M1 with purple stem and M2 with green stem

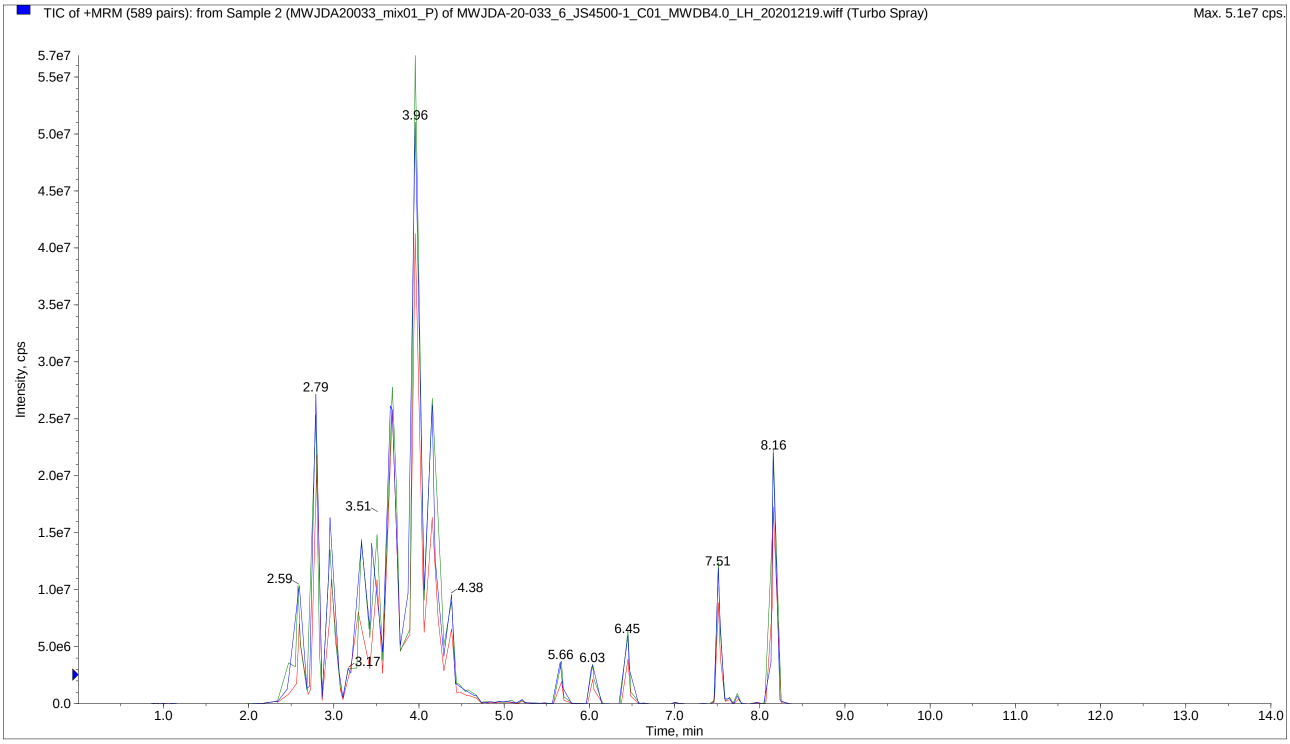

Figure S11: Representative total-ion-chromatogram (TIC) of QC sample

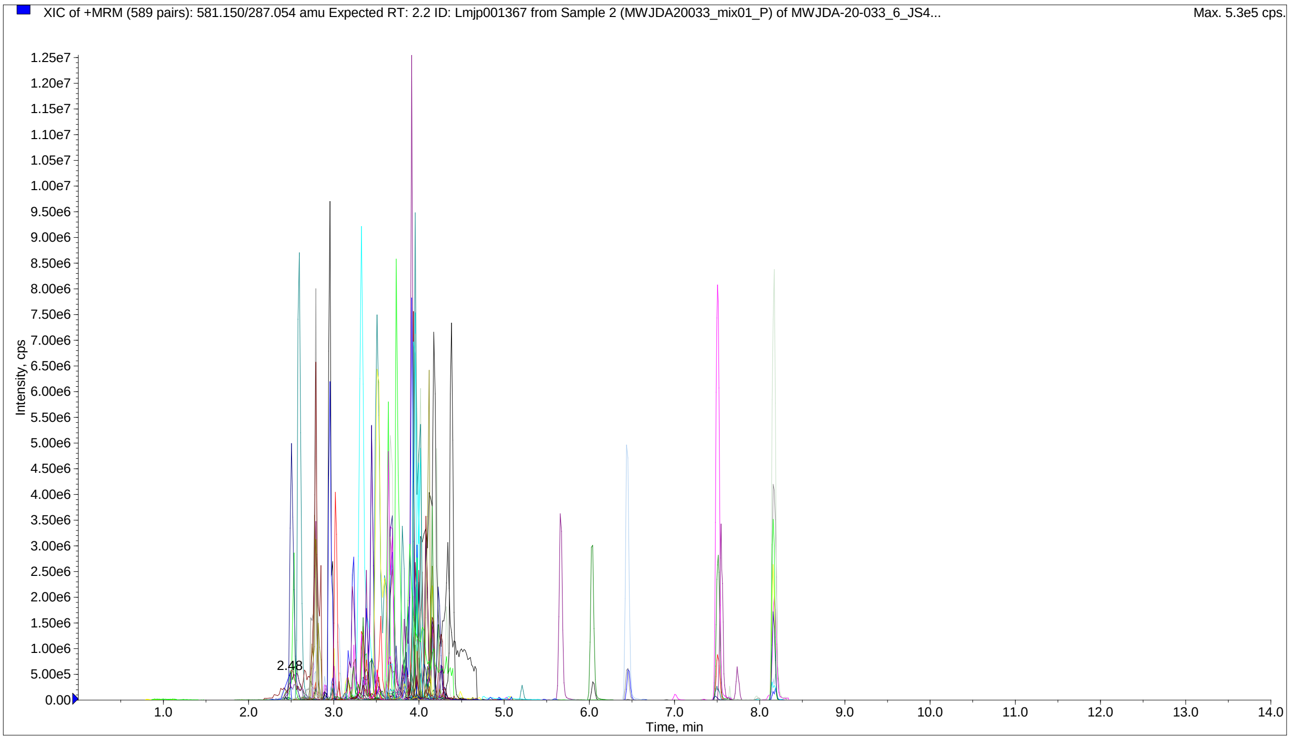

Figure S12: Representative TIC of MRM metabolites detection of QC sample
